# Analyzing and supporting mental representations and strategies in solving Bayesian problems

**DOI:** 10.3389/fpsyg.2026.1642019

**Published:** 2026-03-17

**Authors:** Julia Sirock, Markus Vogel, Tina Seufert

**Affiliations:** 1 Mathematics Education, Institute of Mathematics and Computer Science, University of Education Heidelberg, Heidelberg, Germany; 2 Department for Psychology and Education, Institute for Learning and Instruction, Ulm University, Ulm, Germany

**Keywords:** Bayesian problem-solving, cognitive load, coherence formation, mental model, multiple representations, visualization

## Abstract

Solving Bayesian problems poses many challenges, such as identifying relevant numerical information, classifying and translating it into mathematical formula language, and forming a mental representation. This triggers research on how to support the solving of Bayesian problems. The facilitating effect of using numerical data in frequency format instead of probabilities is well documented, as is the facilitating effect of given visualizations of statistical data. Accordingly, this study examines the differences, in learning success and cognitive load, between the formula, the 2 × 2 table, and the unit square. The visualizations are additionally explained in a descriptive way and created by the participants themselves. The results confirm the hypothesis of the study that learning success is significantly higher when using the unit square and the 2 × 2 table than when using the formula. A contrasting pattern emerged for passive and active load. Significant differences between the unit square and the 2 × 2 table could not be found for learning success and passive and active load. Consequently, the visualization of Bayesian problems, which are explicitly explained and created by the participants, increase solution performance and reduce the effort that the solution of a task requires from the learners.

## 1 Introduction

Bayesian problems refer to situations of uncertainty where inferential judgement is needed. The belonging Bayes Theorem describes the probability of an event, based on prior knowledge of conditions that might be related to the event being of interest. One of the most popular examples is the Mammography problem (Gigerenzer and Hoffrage, 1995; p. 685; adapted from Eddy, 1982):

*The probability of breast cancer is 1% for a woman at age forty who participates in routine screening. If a woman has breast cancer, the probability is 80% that she will get a positive mammography. If a woman does not have breast cancer, the probability is 9.6% that she will also get a positive mammography. A woman in this age group had a positive mammography in a routine screening. What is the probability that she actually has breast cancer? ____%*

In order to calculate the risk of having cancer for this woman, the Bayes Theorem is required. The probability of having cancer with a positive test result *P*(*C*|*T*+) is:


$$\begin{array}{l} P\left(C|T+\right)=\frac{P\left(T+|C\right)\cdot P\left(C\right)}{P\left(T+|C\right)\cdot P\left(C\right)+P\left(T+|\neg C\right)\cdot P\left(\neg C\right)}\\ \;=\frac{0,8\cdot 0,01}{0,8\cdot 0,01+0,096\cdot 0,99}=0,078\;=7,8\% \end{array}$$

The result is counterintuitively low (Eddy, 1982) and research shows a miscalculation of probabilities as well as a lack of understanding the results (McDowell and Jacobs, 2017). Without any further help people only guess the correct answer or try to combine the given numbers in the text without a deeper understanding of the problem.

In this paper we want to address the cognitive processes which are necessary when solving Bayesian problems. Thus, based on theoretical models and empirical findings we first analyze these processes and suggest different instructional approaches to foster them by using multiple representations. The goal of our study is first to substantiate whether these processes are actually crucial while solving Bayesian problems and second to examine the effects of the supporting approaches with regard to the correct solutions of the problems and to the experienced cognitive load.

### 1.1 Bayesian problems require to translate between the mathematical-model world and the real-model world

Central to Bayesian tasks is the translation process from problem statements in the real world into the formula language of the mathematical world which fits the main aspects of modeling in school (cf. Eichler and Vogel, 2015).

Both, the process of identifying the relevant numerical information and the translation into the formula language is particularly difficult, when people cannot grasp the meaning of the numerics, because they are too abstract. One approach to overcome this difficulty is to substitute probabilities in the text with natural frequencies as demonstrated by e.g. Brase (2021); Johnson and Tubau (2015); Gigerenzer and Hoffrage (1995). The Mammography problem in terms of natural frequencies looks like the following:

*100 out of 10,000 women who participate in routine screening have breast cancer. Out of 100 women who participate in routine screening and have breast cancer, 80 will have a positive result. Out of 9,900 women who participate in routine screening and have no breast cancer, 950 will also have a positive result. How many of the women who participate in routine screening and receive a positive test result have breast cancer? Answer: ____ out of ____*

The substitution of probabilities by natural frequencies already represents a translation of the mathematical world into the real world. While probabilities induce a computation, natural frequencies can be observed directly. Via natural frequencies all numerical information are absolutely quantified to a single reference class (i.e., the superordinate set of 10,000 persons), where categories are naturally classified into expected values of the compounded events C∩T+, C∩T−,¬C ∩T+,¬C∩T−. In this case, the conditional distribution does not depend on the between-group (having cancer, not having cancer) base rates, but only on the within-group frequencies (true-positive-rate, false-positive rate). Accordingly, the base rates can be ignored, and the required computations are reduced to a simpler form of B/ayes rule (cf. Johnson and Tubau, 2015). The solution via calculation with natural frequencies would be:


$$P\left(C|T+\right)=\frac{80}{80+950}=7,8\%$$

### 1.2 Bayesian problems use conditioned probabilities

The second challenge when solving Bayesian problems is due to its logical structure and to identify the crucial elements that constitute the problem. The problem structure is one of so-called nested sets, which represent one explanation for the facilitating effect of natural frequencies (Sloman et al., 2003, p. 297). For the above-mentioned example there are 4 subsets of events, defined by the two subsets of each main criteria, i.e. being ill (yes/no) and having a positive test result (yes/no). From a representational perspective, one has to construct a relational mental framework of units that are on a higher level and which can be subdivided into sub-ordinate units. The resulting mental representation thus has a spatial structure. In terms of current cognitive models of knowledge acquisition (e.g., Schnotz and Bannert, 2003) it would be called a depictive mental representation as it is analog to the nested structure of the problem.

According to Mayer's (2014) multimedia principle it could be helpful to provide a depictive representation, i.e. a visualization in addition to the textual problem statement, if learners need to construct a depictive mental representation. However, it is not sufficient to provide any kind of visualization, it should map to the structure of the mental representation (Gentner, 1983). Different studies indicate that visualizations could actually help solving Bayesian problems (Eichler et al., 2020; Brase, 2021, 2008; Khan et al., 2015). Eichler et al. (2020) showed that particularly visualizations that illustrate the nested-sets structure of a Bayesian situation facilitate Bayesian Reasoning.

**Figure 1:**
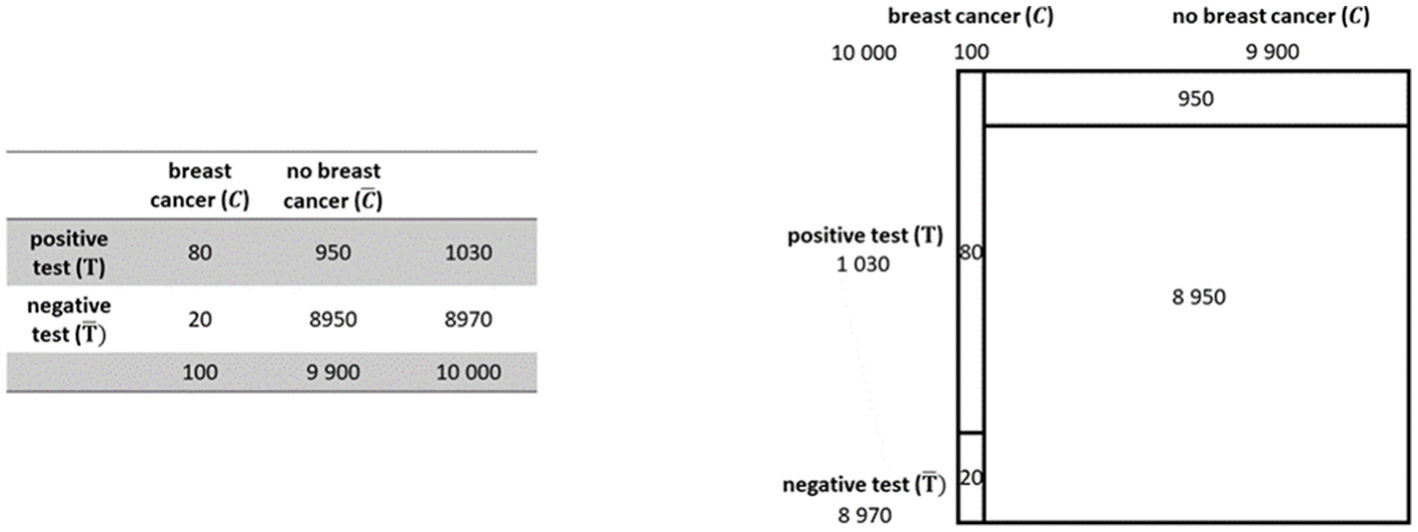
2 × 2 table **(left)** and unit square **(right)** belonging to the mammography problem.

With the visualization by a 2 × 2 table for example, the given information is ordered and thus already pre-structured in columns and rows in such a way that the reader can see at a glance, for example, the positive test rates (see Figure 1). It already contains all values relating to the base rate, number of positive and negative test results, and number of diseases and no diseases but not values such as the false-positive rate. Therefore, it is still up to the reader to bring the elements into relations or to overview covariations.

The unit square is a statistical graph (Tufte, 2015), which means, that the sizes of the partitioned areas are proportional to the sizes of the represented data (see Figure 1). Therefore, the proportions of incidences, like e.g. the base-rate, in a population are represented numerically as well as geometrically. Thus, the numerical descriptively represented information gets an analog depictive counterpart which according to the supplantation effect (Vogel et al., 2007) may lead to a deeper elaboration of the Bayesian situation's mental model via mutual supplementation (cf. above; Schnotz and Bannert, 1999).

Both visualizations turned out to be effective for solving Bayesian problems in terms of an increase in performance (Eichler et al., 2020; Binder et al., 2015). It would also be plausible that it should also be easier to construct the required knowledge representation when provided by an analog representation. However, up to now there is no analysis whether the better match of external and internal representation also affects cognitive load. Taking into account the different aspects of cognitive load (Paas and Van Merriënboer, 1994) one could assume that the visualizations unburden learners from searching for the relevant information and their inter-relation and should thus reduce extraneous load. It might also be possible that the visual ordering and clustering of information within the graph helps to reduce the perceived complexity and thus intrinsic load. Both types of load, extraneous and intrinsic are due to task affordances which learners experience passively. Thus, in a recent paper by Klepsch and Seufert (2021) the required resources are referred to as passive load. In contrast, learners can also decide to invest effort, i.e. they devote resources actively to deal with the task. Thus, these resources are referred to as active load. With regard to the visualizations of Bayesian problems, learners might be activated to use them and consequently, the active load might be increased.

### 1.3 Bayesian problems require to relate different probabilities

One additional requirement when solving Bayesian problems is to apply the Bayes formula. People need to understand what elements determine the denominator and the numerator of the Bayes ratio and what the underlying meaning of this ratio is. The required mental model does not only include single separated elements but their interplay (Schnotz and Bannert, 2003). A mental model is also characterized by its flexibility. Learners can “see” the problem structure and manipulate it mentally (“envisioning” and “running”; Kleer and Brown, 1983, p. 156). With regard to solving Bayesian problems learners could thus be able to specify how the result would change with varying parameters of the problem.

One essential issue of the unit square is that the numerically represented products of conditioning probabilities and conditioned probabilities [e.g., *P*(T+|C) · *P*(C)] which determine the denominator and the numerator of the Bayes formula correspond to the calculation of the rectangular subareas (length multiplied by width) of the unit square. With this kind of area calculation, students are already familiar from basic geometry when learning about conditional probabilities and Bayesian situations. Thus, the in the novices' eyes complex looking Bayesian formula gets potentially better accessible for students because its parts are based on well-known mathematical subroutines. Therefore, the benefit in a mathematical regard is that the unit square can be used to calculate the numerical value of probabilities and to determine the Bayes ratio (cf. Oldford, 2003, p. 1).

### 1.4 Bayesian problems require to deal with multiple representations

Beside the supportive effects of additional visualizations, they also need to be processed. From a multi-representational point of view, like in this case the textual problem statement, the visualization and the formula, dealing with it requires to mentally link the individual representations and build a coherent mental representation; a process called coherence formation (Seufert, 2003).

With regard to the promotion of coherence formation processes, there is a variety of empirical work (see Seufert and Brünken, 2006 for a summary). They show that different approaches are possible to successfully support knowledge acquisition with multiple representations. On the one hand, the improved and deepened processing of individual representations (local coherence formation) is promoted and on the other hand, the actual linking of multiple representations (global coherence formation). Empirical studies show positive effects of prompting learners to find corresponding elements or relations (Schnaubert and Bodemer, 2017; Bodemer and Faust, 2006). However, to support learners in finding these corresponding elements and relations it also turned out to be effective to highlight the correspondences by e.g. color coding (Vogt et al., 2020) or by explicitly explaining relations between representations on a deeper level of understanding (Seufert, 2003). Particularly the combination of highlighting and deep-level help turned out to be supportive in terms of increased learning outcomes and decreased overall cognitive load (Seufert and Brünken, 2006).

## 2 Present study

### 2.1 Hypothesis

The focus of this study is on the comparison of information-equivalent visualizations to support the solution of Bayesian problems, which differ qualitatively with respect to analog and non-analog representation formats. Specifically, the Bayesian formula, the 2 × 2 table and the unit square are examined. The formula itself does not provide any visualized help. The 2 × 2 table, on the other hand, presents the important information from the text in a spatially structured way. Studies already show that the 2 × 2 table supports performance (Eichler et al., 2020; Binder et al., 2015). Based on the additional analogous character of the unit square, the second hypothesis would lead to the assumption that the unit square outperforms the 2 × 2 table. However, (Böcherer-Linder and Eichler 2019) found the unexpected effect that it was the other way round. In their discussion they assume that this unexpected result was partially influenced by the context of a certain item with the most extreme distribution as well as by the unfamiliarity of the unit square, and they argued for further investigations. Using other items in another setting of data collection (within-subject design) including a training how to construct a visualization (cf. below section 2) we argue for the unit square on the theoretical considerations and state:

*Hypothesis 1: Performance will be the highest in tasks that show the unit square for support, followed by tasks that use the 2* × *2 table while in tasks that use the formula the performance is lower*.

Beyond performance, the cognitive load associated with the different visualizations is investigated. Since the 2 × 2 table already has a spatial structure and thus a better correspondence between the external and internal representation, it is assumed that the passive cognitive load is lower compared to the Bayesian formula. Consistent with this, because of the additional analogous nature of the unit square, it is reasonable to assume that passive cognitive load is even lower.

*Hypothesis 2: In tasks that show the unit square for support, passive cognitive load will be lower than in tasks that use the 2* × *2 table and tasks that use the Bayesian formula have the highest passive cognitive load*.

With regard to active cognitive load, it is the other way around - the lower the passive cognitive load, the more focus can be placed on active cognitive load. This leads to the hypothesis:

*Hypothesis 3: In tasks that use the unit square for support, active cognitive load is higher than in tasks that use the 2* × *2 table. The active cognitive load is higher with the 2* × *2 table than with tasks that use Bayes' formula*.

It is also expected that learners' prior knowledge as well as their abilities in spatial and logical reasoning affect the relation between the different visualizations and performance or passive and active load. We therefore assume moderating effects of all these aptitudes: With lower prior knowledge or spatial and logical abilities the differences between the visualizations will be larger than with increasing knowledge and abilities.

*Hypothesis 4 (a-c): Learners' prior knowledge will moderate the relation between the different visualizations and their performance (4a), their passive load (4b) and their active load (4c)*.

*Hypothesis 5 (a-c): Learners' spatial abilities will moderate the relation between the different visualizations and their performance (5a), their passive load (5b) and their active load (5c)*.

*Hypothesis 6 (a-c): Learners' logical abilities will moderate the relation between the different visualizations and their performance (6a), their passive load (6b) and their active load (6c)*.

### 2.2 Participants

A total of 66 psychology and mathematics teaching students took part in the study (*M*_*age*_ = 22,3 years; 75% male) The survey took place in individual interviews via video conference with Zoom in a within-subject design. Both the whiteboard function and recordings of the respective session were used to evaluate the responses.

### 2.3 Material and study design

The study consists of a total of seven typical tasks to capture Bayesian thinking, which were presented using Zoom's whiteboard function. The first task was presented without any assistance to see how the students already deal with the task in terms of prior knowledge. Once students have created their solution to the task, they were provided with step-by-step prompts to develop supporting visualizations. First, the students created a 2 × 2 table by using the prompts, which was then developed into a unit square. At this point the visualizations were created by the students themselves, allowing for deeper understanding (Bodemer et al., 2005).

In a short video with screen recording and voice over, the individual steps are now presented with corresponding numbers of the 2 × 2 table, the unit square and Bayes' formula being highlighted via appropriate color coding. In the video, first the transition of the text task to the representation by a 2 × 2 table is described, then the transition from the 2 × 2 table to the unit square and finally to Bayes' mathematical formula. Thus, at the beginning, you see text and a 2 × 2 table, then a 2 × 2 table and a unit square, and finally all three representations (2 × 2 table, unit square, and mathematical formula) side by side and linked via color coding.

After the video, participants were given six Bayesian tasks with comparable difficulty but different contexts and numbers (two tasks each in similar contexts, for example from the fields of medicine, everyday life, law) and different visualization support (2 × 2 table, unit square, and Bayes formula). Randomization of contexts and order of tasks and visualizations is intended to eliminate bias in the results. Table 1 shows the procedure.

**Table 1:** Summary of the procedure of the study.

**Bayesian task 0 (prior knowledge measure) no visualization context 1**			
**Prompts**			
**Summarizing video**			
**Bayesian Task (visualization)**	**Groups with different contexts per task for randomized order control**		
1 (2 × 2 table)	context 2	context 4	context 6
2 (2 × 2 table)	context 3	context 5	context 7
3 (unit square)	context 4	context 6	context 2
4 (unit square)	context 5	context 7	context 3
5 (Bayes' formula)	context 6	context 2	context 4
6 (Bayes' formula)	context 7	context 3	context 5
	Control variables (course of study, Abitur grade in maths, test for spatial and logical thinking)		

Following each task, active and passive cognitive load were measured by self-report as dependent variables using the two items “I exerted myself.” (Active cognitive load) and “It was exhausting.” (Passive cognitive load; cf. Klepsch and Seufert, 2021). The items were determined on a 5-point Likert scale (1 = “Does not apply at all” to 5 = “Applies completely”).

Performance as another dependent variable was determined by the solution quality of the six Bayesian tasks. For this purpose, one point each was awarded for the subsample of the numerator and the denominator of the fraction of the Bayes' formula to be determined and one point for the correct relation. Performance for the first, unassisted task was calculated in the same way and was used as prior knowledge measure.

As additional control variables, the course of study and Abitur grade in math will be inquired about. The course of study was surveyed for descriptive purposes only. Furthermore, learners' verbal, numerical and figural thinking abilities were analyzed by using the IST-Screening subtests by Liepmann et al. (2012). The ability score is the percent of correctly solved tasks in these tests. The students‘ prior knowledge is already assessed via the first task.

### 2.4 Deviation from preregistration

Since this is a preregistered report, we will briefly discuss here the points that deviated from the preregistration and why.

We made a total of four deviations, the first of which concerns the sample. Originally, only first-semester students were to be surveyed. However, as this restriction would have resulted in a significantly smaller sample and thus made it impossible to evaluate the data in a meaningful way in some cases, we also allowed students from higher semesters to participate, but still with a focus on psychology or mathematics teaching.

Due to a better fit with our study, a different test instrument than originally specified was used to assess numerical and figural thinking abilities – originally, it was planned to use the KFT cognitive abilities subtests for paper folding and numerical series by Heller and Perleth (2000), but now the IST screening subtests by Liepmann et al. (2012) were used. However, this second deviation has no impact on the evaluation of the study.

A third deviation relates to missing values: there are hardly any missing values, so we did not perform any imputation for the evaluation.

The last deviation refers to the solving process: Originally, think aloud data was to be collected here. Although the participants were asked to think aloud at the beginning, it was not insisted that they actually do so. As a result, the participants were restrained and we only gained little think aloud data and therefore no specific evaluation of it. However, it quickly became clear during the surveys that the behavioral data provided valuable insights even without think aloud, so the participants were not repeatedly asked to think aloud.

### 2.5 Data analysis

All participants were given the same tasks so that the analyses can all be done within subject by repeated measures ANOVAs with subsequent contrast analyses. All statistical analyses were performed with SPSS (IBM SPSS Statistics 29).

First, the descriptive statistics were calculated for the dependent variables across all conditions. In order to investigate differences between the conditions, ANOVAs were performed with the learning success, the active load and the passive load as dependent variables. Furthermore, contrast analyses were performed for learning success as well as for passive and active load. The significance level for all calculations was *p* < 0.05.

### 2.6 Results

#### 2.6.1 Performance in Bayesian tasks

**Figure 2:**
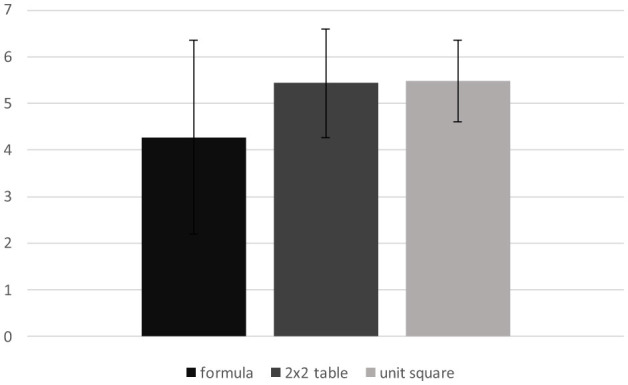
Distribution of points for the three visualizations.

First, the descriptive statistics for the three different experimental conditions (formula, 2 × 2 table, unit square) were calculated. As there are two tasks with three possible points each, a maximum score of six points is achievable. There was already a clear difference between the formula condition (*M* = 4.28, *SD* = 2.08), the 2 × 2 table condition (*M* = 5.46, *SD* = 1.16) and the unit square condition (*M* = 5.48, *SD* = 0.89), as illustrated in Figure 2.

An ANOVA with repeated measures was performed to gain further insight. As the Mauchly test shows violation of sphericity, Greenhouse-Geisser correction was used [Mauchly-W(2) = 0.701, *p* < 0.001]. A repeated measures ANOVA with a Greenhouse-Geisser correction determined that there was a significant difference between at least two of the three conditions [*F*_(1, 54;97, 01)_ = 18.319, *p* < 0.001, partial η^2^ = 0.225, 95.%-CI[0.0898, 0.3520]].

The contrast analyses revealed a significant difference between the 2 × 2 table [*M* = 5.46, *SD* = 1.16] compared to the formula [*M* = 4.28, *SD* = 2.08] of −1.434 (SE = 0.378), Bonferroni-adjusted *p* < 0.001, partial η^2^ = 0.186, 95.%-CI[0.0431, 0.3448], as well as between the unit square [*M* = 5.48, *SD* = 0.89] compared to the formula of −1.841 (*SE*=.339), Bonferroni-adjusted *p* < 0.001, partial η^2^ = 0.319, 95.%-CI[0.1391, 0.4701]. However, no significant effect was found between the 2 × 2 table and the unit square [−0.407, SE = 0.221, Bonferroni-adjusted *p* = 0.140, partial η^2^ = 0.051, 95%-CI[0.0000, 0.1837]].

The control variables prior knowledge, verbal, numerical and figural thinking abilities were analyzed as moderators in individual moderation analyses via the PROCESS plug-in of Hayes (2022) in SPSS. In PROCESS, moderation is determined via the ΔR^2^ when adding the interaction term; this model comparison underlies all reported moderation results. It uses ordinary least squares regression, yielding unstandardized coefficients for all effects. Bootstrapping with 5,000 samples was employed to compute the confidence intervals.

First, a moderation analysis was run to determine whether the interaction between prior knowledge and type of visualization predicts performance in Bayesian Tasks. The overall model was significant, *F*_(3, 187)_ = 14.55, *p* < 0.001, predicting 43.50% of the variance. Moderation analysis showed that prior knowledge moderated the effect between visualization and performance in Bayesian Tasks significantly, ΔR^2^ = 3.04%, *F*_(1, 187)_ = 7.00, *p* = 0.009. The model with interaction is therefore significantly better than the model without interaction and explains 3.04% more of the variance of the dependent variable in comparison.

Second, a moderation analysis was run to determine whether the interaction between verbal thinking abilities and type of visualization predicts performance in Bayesian Tasks. The overall model was significant, *F*_(3, 187)_ = 16.37, *p* < 0.001, predicting 45.60% of the variance. Moderation analysis showed that verbal thinking abilities did not moderate the effect between visualization and performance in Bayesian Tasks significantly, *F*_(1, 187)_ = 0.43, *p* = 0.516.

Third, a moderation analysis was run to determine whether the interaction between numerical thinking abilities and type of visualization predicts performance in Bayesian Tasks. The overall model was significant, *F*_(3, 187)_ = 7.35, *p* < 0.001, predicting 32.48% of the variance. Moderation analysis showed that numerical thinking abilities moderated the effect between visualization and performance in Bayesian Tasks significantly, ΔR^2^ = 3.16%, *F*_(1, 187)_ = 7.47, *p* = 0.007.

At least a moderation analysis was run to determine whether the interaction between figural thinking abilities and type of visualization predicts performance in Bayesian Tasks. The overall model was significant, *F*_(3, 187)_ = 10.59, *p* < 0.001, predicting 38.12% of the variance. Moderation analysis showed that figural thinking abilities did not moderate the effect between visualization and performance in Bayesian Tasks significantly, *F*_(1, 187)_ = 3.23, *p* = 0.074.

In summary, there appears to be a difference between the two graphical visualizations (2 × 2 table and unit square) and the formula in the descriptive data, which is confirmed once again by the ANOVA with contrast analyses. Furthermore, moderation analyses show significant results regarding the interaction between prior knowledge and type of visualization for predicting performance in Bayesian Tasks as well as between numerical thinking abilities and type of visualization for predicting performance in Bayesian Tasks.

#### 2.6.2 Passive load

**Figure 3:**
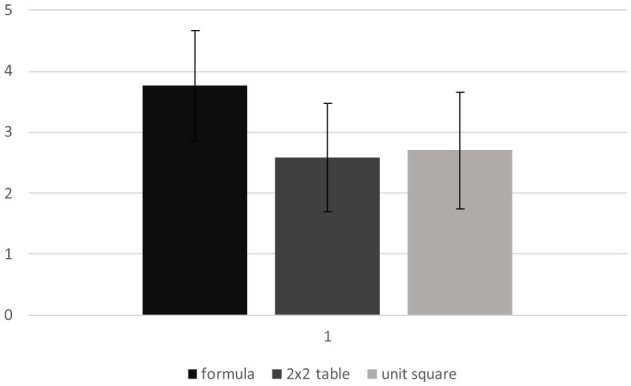
Passive load regarding the three visualizations, measured on a 5-point Likert scale.

According to the previous analysis, the descriptive statistics for the three different experimental conditions (formula, 2 × 2 table, unit square) were calculated. As illustrated in Figure 3, there was already a clear difference between the active load of the formula condition (*M* = 3.76, *SD* = 0.910), the 2 × 2 table condition (*M* = 2.59, *SD* = 0.891) and the unit square condition (*M* = 2.71, *SD* = 0.962).

An ANOVA with repeated measures was performed to gain further insight. As the Mauchly test does not show any violation of sphericity, no adjustment was used [Mauchly-W(2) = 0.983, *p* = 0.588]. A repeated measures ANOVA determined that there was a significant difference between at least two of the three conditions [*F*_(2, 124)_ = 58.464, *p* < 0.001, partial η^2^ = 0.485, 95%-CI[0.3553, 0.5760]].

Similar to the previous contrast analysis, it revealed a significant difference between the 2 × 2 table [*M* = 2.59, *SD* = 0.891] compared to the formula [*M* = 3.76, *SD* = 0.910] of 1.171 (*SE* = 0.124), Bonferroni-adjusted *p* < 0.001, partial η^2^ = 0.591, 95.%-CI[0.4247, 0.6928], as well as between the unit square [*M* = 2.71, *SD* = 0.962] compared to the formula of −1.044 (*SE* = 0.122), Bonferroni-adjusted *p* < 0.001, partial η^2^ = 0.543, 95.%-CI[0.3670, 0.6556]. However, no significant effect was found between the 2 × 2 table and the unit square [−0.127, *SE* = 0.111, Bonferroni-adjusted *p* = 0.513, partial η^2^ = 0.021, 95%-CI[0.0000, 0.1314]].

As before, the control variables prior knowledge, verbal, numerical and figural thinking abilities were analyzed as moderators in individual moderation analyses via the PROCESS plug-in of Hayes (2022) in SPSS. It uses ordinary least squares regression, yielding unstandardized coefficients for all effects. Bootstrapping with 5,000 samples was employed to compute the confidence intervals.

First, a moderation analysis was run to determine whether the interaction between prior knowledge and type of visualization predicts passive load. The overall model was significant, *F*_(3, 183)_ = 15.18, *p* < 0.001, predicting 44.64% of the variance. Moderation analysis showed that prior knowledge did not moderate the effect between visualization and passive load significantly, *F*_(1, 183)_ = 1.205, *p* = 0.274.

Second, a moderation analysis was run to determine whether the interaction between verbal thinking abilities and type of visualization predicts active load. The overall model was significant, *F*_(3, 183)_ = 14.34, *p* < 0.001, predicting 43.63% of the variance. Moderation analysis showed that verbal thinking abilities did not moderate the effect between visualization and passive load significantly, *F*_(1, 183)_ = 0.272, *p* = 0.603.

Third, a moderation analysis was run to determine whether the interaction between numerical thinking abilities and type of visualization predicts passive load. The overall model was significant, *F*_(3, 183)_ = 13.16, *p* < 0.001, predicting 42.12% of the variance. Moderation analysis showed that numerical thinking abilities did not moderate the effect between visualization and passive load significantly, *F*_(1, 183)_ = 0.307, *p* = 0.580.

At least a moderation analysis was run to determine whether the interaction between figural thinking abilities and type of visualization predicts active load. The overall model was significant, *F*_(3, 183)_ = 14.20, *p* < 0.001, predicting 43.46% of the variance. Moderation analysis showed that figural thinking abilities did not moderated the effect between visualization and active load significantly, *F*_(1, 183)_ = 1.18, *p* = 0.280.

In summary, there appears to be a difference in passive load between the two graphical visualizations (2 × 2 table and unit square) and the formula in the descriptive data, which is confirmed once again by the ANOVA with contrast analyses. Furthermore, moderation analyses show no significant results.

#### 2.6.3 Active load

**Figure 4:**
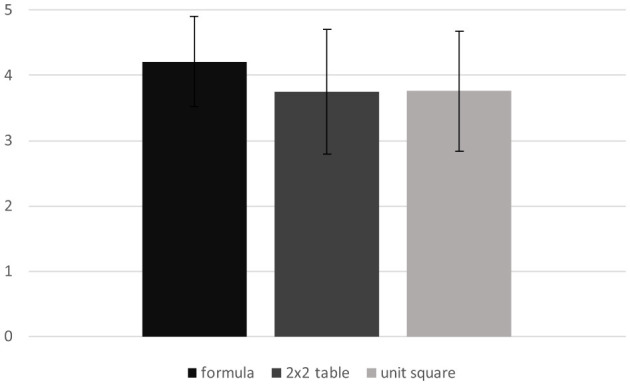
Active load regarding the three visualizations, measured on a 5-point Likert scale.

Similar to the analysis for the performance, the descriptive statistics for the three different experimental conditions (formula, 2 × 2 table, unit square) were calculated with regard to active load. As illustrated in Figure 4, there was already a clear difference between the active load of the formula condition (*M* = 4.21, *SD* = 0.694), the 2 × 2 table condition (*M* = 3.76, *SD* = 0.967) and the unit square condition (*M* = 3.79, *SD* = 0.897).

An ANOVA with repeated measures was performed to gain further insight. As the Mauchly test shows violation of sphericity, Huynh-Feldt correction was used [Mauchly-W(2) = 0.775, *p* < 0.001]. A repeated measures ANOVA with a Huynh-Feldt correction determined that there was a significant difference between at least two of the three conditions [*F*_(1.67, 103.57)_ = 14.000, *p* < 0.001, partial η^2^ = 0.184, 95.%-CI[0.0625, 0.3054]].

Just as in the previous contrast analysis, it revealed a significant difference between the 2 × 2 table [*M* = 3.76, *SD* = 0.967] compared to the formula [*M* = 4.21, *SD* = 0.694] of 0.452 (*SE* = 0.114), Bonferroni-adjusted *p* < 0.001, partial η^2^ = 0.204, 95%-CI[0.0528, 0.3639], as well as between the unit square [*M* = 3.79, *SD* = 0.897] compared to the formula of −0.429 (*SE* = 0.098), Bonferroni-adjusted *p* < 0.001, partial η^2^ = 0.236, 95%-CI[0.0737, 0.3953]. No significant effect was found between the 2 × 2 table and the unit square [−0.024, *SE* = .073, Bonferroni-adjusted *p* > 0.999, partial η^2^ = 0.002, 95%-CI[0.0000, 0.0700]].

Again, the control variables prior knowledge, verbal, numerical and figural thinking abilities were analyzed as moderators in individual moderation analyses via the PROCESS plug-in of Hayes (2022) in SPSS. It uses ordinary least squares regression, yielding unstandardized coefficients for all effects. Bootstrapping with 5,000 samples was employed to compute the confidence intervals.

First, a moderation analysis was run to determine whether the interaction between prior knowledge and type of visualization predicts active load. The overall model was significant, *F*_(3, 183)_ = 5.67, *p* = 0.001, predicting 29.17% of the variance. Moderation analysis showed that prior knowledge did not moderate the effect between visualization and performance in Bayesian Tasks significantly, *F*_(1, 183)_ = 0.0021, *p* = 0.963.

Second, a moderation analysis was run to determine whether the interaction between verbal thinking abilities and type of visualization predicts active load. The overall model was not significant, *F*_(3, 183)_ = 3.34, *p* = 0.021.

Third, a moderation analysis was run to determine whether the interaction between numerical thinking abilities and type of visualization predicts active load. The overall model was not significant, *F*_(3, 183)_ = 3.77, *p* = 0.012.

At least a moderation analysis was run to determine whether the interaction between figural thinking abilities and type of visualization predicts active load. The overall model was significant, *F*_(3, 183)_ = 5.83, *p* < 0.001, predicting 29.54% of the variance. Moderation analysis showed that figural thinking abilities did not moderated the effect between visualization and active load significantly, *F*_(1, 183)_ = 0.01, *p* = 0.930.

In summary, there appears to be a difference in active load between the two graphical visualizations (2 × 2 table and unit square) and the formula in the descriptive data, which is confirmed once again by the ANOVA with contrast analyses. Furthermore, moderation analyses show no significant results.

#### 2.6.4 Qualitative analyses of the recordings

A review of the videos shows that the participants mainly solved the tasks mentally with the help of the information provided and, in some cases, with the help of their own notes, while verbal expressions are hardly present. Overall, the recordings offer valuable insights into the solution process, which are presented below.

Even when solving the initial task (prior knowledge), it is noticeable that the majority of the participants (46 out of 66) make notes of various kinds. The notes often contain a compilation of the (relevant) information. Some participants (9 out of 45 with notes) create a tree diagram, another 9 participants make notes in the form of (conditional) probabilities and only one person uses a 2 × 2 table to solve the task. The remaining notes could not be assigned to a specific visualization, here the relevant data was simply written out.

Almost all participants (64 out of 66) were able to independently create a 2 × 2 table using the prompts following the initial task. A similar picture emerged when creating the unit square using the prompts: 58 out of 66 participants were able to successfully create a unit square. Some of the participants corrected their solution to the initial task in the course of creating the 2 × 2 table or unit square.

In the following six Bayesian tasks, which had to be solved by the participants using a given visualization (formula, 2 × 2 table or unit square), it became apparent that some of the participants preferred a different visualization. This was expressed by drawing a different visualization than the one already given. In particular, in addition to the given formula, the 2 × 2 table was written down (in 28 of 132 cases in which the formula was given). Few other visualization changes were observed: in 7 of 132 cases, the formula was noted when the 2 × 2 table was given, and in 5 cases, the formula was noted when the unit square was given. In addition, notes were made in 17 of 132 cases when the formula was given, and in 1 of 132 cases, notes were made when the 2 × 2 table was given and one person took notes for each task. The notes could not be assigned to any other visualization. No other visualization changes occurred.

### 2.7 Discussion

The aim of this research paper is to investigate the influence of different types of visualization aids, combined with educational psychology approaches, on learning success, active and passive load for in-depth understanding. To this end, the results are first summarized, interpreted and placed in the context of previous research. The limitations of the methodology and the limitations of this study are then discussed in more detail. The implications for science and practice are followed by a general conclusion and an outlook for the future, which will round off this work.

#### 2.7.1 Interpretation and classification of the results

##### 2.7.1.1 Learning success

The present study clearly showed that visualizations have a considerable influence on learning success compared to the formula. The sequence of effective support formulated in Hypothesis 1 is evident in the descriptive data, but not all differences are significant: the solution performance was higher when the tasks were visualized with the unit square than when the 2 × 2 table was used. The lowest learning success rate was recorded for tasks in which the formula was given as an aid. However, there was only a statistically relevant difference between the visualization conditions compared to the formula.

These results confirm the assumption that the visualizations of the 2 × 2 table and the unit square contribute to a higher solution performance in comparison to the formula (Binder et al., 2015; Böcherer-Linder and Eichler, 2019; Garcia-Retamero and Hoffrage, 2013). In addition, the educational psychological considerations that pictorial representations contribute to better understanding compared to non- pictorial learning aids are also supported (Mayer, 2014). In particular, the results confirm the hypothesis underlying this research that the unit square and the 2 × 2 table in combination with deep structure aids and surface cues such as creating one's own visualizations or color coding in the beginning, have a positive influence on learning success in solving Bayesian problems. Furthermore, the graded effect could be proven that the learning success with the unit square is the highest, followed by the learning success with the 2 × 2 table and the lowest solution performance with the formula, even if the effect between the unit square and the 2 × 2 table remains descriptive and does not become significant. This also fits with the assumptions already made by Vogel et al. (2019) that the unit square is a particularly effective learning aid due to its pictorial character and the proportional representation of ratios. One reason for the non-significant difference in solution performance between the 2 × 2 table and the unit square could be that the participants are more familiar with the 2 × 2 table than with the unit square due to mathematics lessons at school and statistics lectures in psychology or mathematics studies. Accordingly, they have more practice with this type of visualization. According to Seufert (2003), training with multiple representations could have influenced the learning success with the corresponding learning aid.

It could also play a role in mental representation that the participants were presented with the visualizations in a specific order at the beginning (both when creating them themselves and in the video). In both cases, a 2 × 2 table was first created based on the task, and a unit square was then constructed on this basis. It is possible that the participants continue to perform these transitions in sequence, which could have an impact on the use of the visualizations and, if so, on performance.

##### 2.7.1.2 Passive load

The results for the passive load also support hypothesis 2, according to which the effort required by a person to solve a task depends on how the assistance given for the task is designed. However, it is important to note that there is also a difference between the visualization conditions compared to the formula. This was shown by significant results between formula and unit square and formula and 2 × 2 table in the contrast analyses. This means, that the passive load in tasks with the unit square and with the 2 × 2 table is less demanding for the learner compared to tasks with the formula. The contrast between the unit square and the 2 × 2 table was not statistically significant.

In general, the results support earlier findings that prove that extraneous load can be influenced by suitable visualizations (Mayer, 2014). In this study, the mental resources that a task demands from learners were summarized as passive cognitive load according to Klepsch and Seufert (2021). Passive cognitive load can therefore be reduced through suitable visualization. This effect can also be used in mathematics didactics, which is also confirmed by the present study. One reason why the passive load of the 2 × 2 table does not differ significantly from that of the unit square could lie in the global coherence that should be achieved with the prompts and the explanatory video (Seufert and Brünken, 2006). According to this, the participants could have understood the connection between the questions, the 2 × 2 table and the unit square so well that they perceive the tasks with the 2 × 2 table as similarly strenuous as those with the unit square.

##### 2.7.1.3 Active load

Contrary to the assumptions from hypothesis 3, the descriptive statistics show a high active load in all conditions. In particular, the active load for the formula is significantly higher than for the other visualizations. This was shown in significant results between formula and unit square and formula and 2 × 2 table in the contrast analyses. No significant difference was found between 2 × 2 table and unit square.

In view of the relatively high average active load in all conditions, it can primarily be concluded that all participants invested mental effort when completing the tasks, regardless of the visualization formats (Seufert, 2018). One explanation for this could be the relationship between the intrinsic load and the germane load, as described by Paas et al. (2010). They postulate that the “Germane Load” refers to the resources of the working memory that are used to deal with the “Intrinsic Load”. If the “intrinsic load” changes - i.e. the complexity of the task - the “germane load” adapts accordingly. In this study, the tasks were standardized. Although the numbers changed, the influencing variables, the question and answer format, and the natural frequencies remained identical across all tasks in order to maintain internal validity and prevent varying levels of task difficulty. It can therefore be assumed that the “intrinsic load” also remained constant across all tasks. Nevertheless, learners invested more effort when working with the formula. As the passive load results show this was the most strenuous condition and hence learners might have tried to compensate the increased demands with higher investment. Another explanation could be seen in the fact that learners should work with the formula, but as the qualitative results show they additionally used the visual formats. Hence they needed an increased investment. They seem to have followed the goal to succeed and might be highly motivated. Therefore, in future studies it would be beneficial to analyze learners motivation.

##### 2.7.1.4 Qualitative analyses

Even the simple observation of the participants when solving the initial task is very revealing: many participants first made their own notes in order to collect the information. This confirms the relevance of visualizations and structured presentations of key information. This relevance was also evident later on. While the test subjects created the 2 × 2 table and the unit square themselves with the help of the prompts, some of them were already able to correct their original solution to the initial task immediately.

Almost all participants were able to create the 2 × 2 table and the unit square using the prompts. On the one hand, this confirms the validity of the prompts used and, on the other hand, it shows that both the 2 × 2 table and the unit square can be created in a short time with just a few instructions.

Last but not least, the frequent use of the 2 × 2 table instead of the formula already given shows that its clear presentation of the relevant information is preferred. This indicates that the 2 × 2 table is perceived as more catchy and helpful than the formula. At this point, the question naturally arises as to why a unit square is not drawn. There are two possible arguments: on the one hand, the unit square is currently less well known, as it has not yet found its way into the German curriculum. On the other hand, it is possible that when creating the unit square, too much perfection is sought in terms of exact proportions, which leads to both a kind of fear of inaccuracy and a high consumption of time.

This raises further research questions that should be examined in detail in subsequent studies.

#### 2.7.2 Limitations of the methodology

A very high average was recorded for learning success as well as for active load across all conditions. Combined with the significant effects of the contrast analyses, these could be ceiling effects. The tasks therefore might have been too easy. The same argument can be applied to the floor effects with passive load (Becker-Mrotzek and Grabowski, 2022). The tasks for Bayes' theorem were all set uniformly, which is why the aspect of ceiling effects in learning success and floor effects in passive load must be taken into account when interpreting the results. The clear gradations of the mean values in the descriptive results in learning success and passive and active load nevertheless demonstrate the justified assumption of a linear trend.

Despite the absence of a normal distribution, ANOVAs were used for the calculation. Due to the repeated measures design and the standardized procedure, the study has a high internal validity, which justifies the use of a robust method such as ANOVA (Schmider et al., 2010). In addition, the large effect sizes provided a sample-independent measure of the influence of visualization aids on the dependent variables. However, the relatively homogeneous sample harbors further aspects that should be considered when interpreting and replicating this study.

#### 2.7.3 Limitations of the study

The limited sample of young psychology and mathematics teaching students not only affects methodological aspects of the study, but also limits the generalizability of the results. Combined with the highly standardized procedure, this means that the results cannot be transferred to other populations, such as those with a lower educational status or a higher average age. The external validity is therefore limited. Nevertheless, it should also be mentioned at this point that the standardized procedure ensures internal validity and thus justifies the procedure on which this study is based.

Another factor that should be considered when evaluating the results is that the participants were observed while solving the tasks and answering the items on active and passive load. Although the test subjects switched off their camera and microphone during the calculation phase, observational effects could have influenced the results. On the one hand, it is possible that the participants were more nervous due to the feeling of being observed (Zajonc, 1965). On the other hand, the sole presence of another person could also have made them try harder to solve the tasks correctly (Dashiell, 1935). However, since the aim of the study was to illustrate differences between the visualization conditions and the observation was uniform across all tasks and participants, it is unlikely that this possible confounding variable had a relevant influence on the hypothesis investigated regarding the effect of visualization on learning success.

Another point to note is the possible tendency to give socially desirable answers, especially regarding active load. Since the item was used to indicate the effort invested in solving the task, it is possible that the answers were placed rather high in order to signal to the test administrator that the task had been solved conscientiously (Schneider and Hasselhorn, 2008). The feeling of being observed while ticking could have reinforced this effect. The explanation about the anonymization of the data, as well as the instructions to mark the questions truthfully, should counteract this effect.

Finally, it must be noted that the studies deal specifically with Bayesian problems and the findings cannot be easily transferred to other areas of mathematics. Nevertheless, the study represents an important step toward investigating the influence of visualizations on performance as well as passive and active load. Based on this research work, there are therefore some important implications for science in order to investigate this.

#### 2.7.4 Implications for science and further research questions

An obvious research design to demonstrate the effects of educational psychology approaches would be to investigate the difference in learning success, passive and active load between an intervention group and a control group. An intermediate subject factor would thus be added to the present study. The experimental group would receive the instructional part of this study and then solve the tasks with given visualization aids. In the control group, the prompts and the explanatory video would be omitted. This would result in a 2 × 3 repeated measures design in which both the main effects of the educational psychology approaches and the visualizations as well as the interaction effects could be investigated.

In order to ensure the transferability of the results of this study to other populations, it would also be important to replicate the study with a more heterogeneous sample. Attention should be paid to varying levels of education and age groups. At this point, it would be particularly interesting to see whether there would be significant effects of the control variables collected in this study on learning success and active and passive load in a more heterogeneous sample.

Another aspect is to compare the effects of the unit square and the 2 × 2 table combined with explanations of correlations and self-creation of learning material with other types of visualization of Bayesian problems. Accordingly, visualization methods that have also proven promising in research, such as (double) tree diagrams (Wassner et al., 2002), could be combined with educational psychology approaches to convey a global understanding. It would be interesting to see whether the unit square and the 2 × 2 table would also be as clearly superior to these as the formula.

Due to the possible ceiling and floor effects in the study, another important question would be whether the level of difficulty of the tasks has an effect on learning success and active and passive load. On the basis of this study, it can be stated that visualization and explanation have an influence on learning success with the same level of difficulty of the tasks. How this relationship changes depending on the complexity of the task is another interesting subject of investigation. It would also be a way to better understand the relationship between active load and solving conditional probabilities. As already explained, the degree of difficulty influences a person's intrinsic load. Assuming that the active load is closely linked to the intrinsic load (Paas et al., 2010), a varying degree of difficulty of the tasks would have a considerable influence on the active load, unlike in this study.

#### 2.7.5 Implications for practice

The results of this study highlight that suitable visualizations can support Bayesian problem solving. The combination of the explanation of direct correlations and the self-creation of learning material may have a positive effect on learning success and the effort required to solve such a task. Based on this, there are some important consequences for practice.

First of all, we need to take a look at the educational system. It would contribute to a comprehensive understanding of conditional probabilities if they were already presented clearly in math lessons at school. Based on this study, the unit square and the 2 × 2 table in particular have proven to be very effective. It is also important to explain the direct relationships between relevant influencing variables in Bayesian problems. The learning material should also be created by the students themselves and designed in such a way that important figures are easy to recognize and classify. The lessons should therefore aim to convey a holistic understanding of how the relevant variables are related to each other, how they can be clearly visualized and which values in which ratio in the visualizations lead to the final result. An overarching goal would be to implement these strategies consistently across the curriculum. In addition, the approaches to teaching Bayesian problems should not be limited to school, but should be transferred to subsequent further education, such as vocational training and university studies.

Especially for professional fields that are confronted with conditional probabilities, it would be important to provide a good overview of Bayesian problems during training. One way to implement this would be to offer compulsory modules during the course of study (e.g. medicine, law, psychology, etc.) in which the strategies described above are applied in order to convey the topic of conditional probabilities and their importance in later professional life (cf. Büchter et al., 2022). Further education and training courses on this topic could also be useful for already trained specialist staff, in which specific relationships are explained again using visualizations.

To go one step further, the dissemination of visualizations and explanations of Bayesian statistics in the media could also be helpful. Both in the digital and analog press, this could lead to a better understanding of these facts among the general public. Especially in the case of socially relevant topics or crises, such as the Covid-19 pandemic, it would be essential to visualize and explain this content in such a way that a global understanding of relevant influencing factors can be guaranteed.

The long-term goal would therefore be to visualize and explain Bayesian problems in a coherent way in many areas, such as schools, education and training, professional life and media. So that, on the one hand, our society understands the logic behind Bayesian problems at an early stage as part of its education and, on the other hand, an understanding is achieved among population groups that have not come into contact with conditional probabilities in their school and further education careers through visualizations and explanations.

## 3 Conclusion and outlook

In summary, the results of this study show that visualizations such as the 2 × 2 table and the unit square support learners in solving Bayesian problems and are associated with higher performance compared to formulas. Also, the combination of explanations of direct correlations and the self-creation of learning material in the beginning of the study may have a positive effect on the learning success and the required effort of the tasks. The findings confirm central theoretical assumptions and the overarching hypotheses of this study. How the active load behaves with regard to visualizations in combination with explanations and self-creation of learning material requires further research. The present study contributes to stimulating further scientific research on this topic. It thus contributes to spreading a generally better understanding of Bayesian problems in our society so that in the future, even in crises such as the Covid-19 pandemic, there will be fewer misunderstandings, misinterpretations and false reports regarding conditional probabilities.

## Data Availability

The raw data supporting the conclusions of this article will be made available by the authors, without undue reservation.

## Appendix A: Prompts for creating 2 × 2 table and unit square

Each prompt was read aloud to the participants up to twice.

**Prompts for creating the 2** **×2 table**

**Prompt 1:**

You now have an empty 2 × 2 table here. Many people find it helpful to enter the two characteristics and their manifestations, in our case breast cancer and the test result, into this 2 × 2 table. Try it out.

**Prompt 1 (further help):**

Try entering the two characteristics of breast cancer, i.e., sick and healthy, in the top row, and enter the test results, i.e., positive or negative, in the left column. Alternative Prompt 1 (further help; if participant has swapped two cells): You have almost filled in the 2 × 2 table correctly. You have only swapped two fields. Take another close look at the task and pay attention to (name the feature/property that has been swapped).

**Prompt 2:**

Now you can assign the numbers you find in the text of the task to the properties in the four cells. This will allow you to calculate and enter the marginal frequencies as well.

**Prompt 2 (further help):**

In the text, you will find, for example, the number of people who have breast cancer and have tested positive. In our case, that is 80 people. Where could you enter this number in your table? Try to fill in the other cells of the table using this scheme and then calculate the marginal frequencies. Note that the table field covers both characteristic values “suffering from breast cancer” and “positive test result”.

**Prompts for creating the unit square**

**Prompt 1:**

Now let's create a unit square together. The goal is to end up with a square in which the groups of different sizes occupy more or less space depending on their size. To do this, we will now only look at the inner part of the 2 × 2 table, i.e., the 10,000 people who were tested. The vertical line here separates the healthy women from the sick women. Now move the vertical line so that the area shows how many women are healthy in relation to the women who are sick.

Now look only at the right side of the unit square, i.e., all healthy women. Now move the first horizontal line so that the area shows the ratio of healthy women who tested positive and negative. Then look only at the left side of the unit square, i.e., the sick women. Now move the horizontal line so that the area shows the ratio of sick women who tested positive and negative.

**Prompt 1 (further help):**

I'll guide you through it step by step again. First, we move the vertical line so that the ratio of sick to healthy people becomes clear. In our example, this means that the healthy people must occupy significantly more space than the sick people.

Once you have done this, first look only at the right side of the unit square, i.e. all healthy individuals, in our example the 950 and the 8,950. Move the line so that the area shows that the 8,950 individuals who tested negative are almost 10 times more than the 950 who tested positive. Then look only at the left side of the square, i.e., the sick people in our example, the 20 and the 80. Move the line here so that the area shows that the 80 who tested positive are about 4 times more than the 20 who tested negative.

**Prompt 2:**

Now, as in the 2 × 2 table, add the marginal frequencies next to the square.

**Prompt 2 (further help):**

Take another look at the 2 × 2 table. At the end of the second row, for example, you will find the total number of people who tested positive. That is 1030 in total. Think again about where you could best write this number at the edge of the unit square. Do the same for the remaining frequencies at the end of the row or column.
